# Simple Bedside Predictors of Survival after Percutaneous Gastrostomy Tube Insertion

**DOI:** 10.1155/2019/1532918

**Published:** 2019-11-16

**Authors:** Wisam Sbeit, Anas Kadah, Amir Mari, Mahmud Mahamid, Tawfik Khoury

**Affiliations:** ^1^Department of Gastroenterology, Galilee Medical Center, Nahariya, Israel; ^2^Faculty of Medicine in the Galilee, Bar-Ilan University, Safed, Israel; ^3^Gastroenterology and Endoscopy Units, EMMS Nazareth Hospital, Nazareth, Israel; ^4^Gastroenterology Department, Shaare Zedek Medical Center, Jerusalem, Israel

## Abstract

**Background:**

Percutaneous endoscopic gastrostomy (PEG) tube insertion is an increasingly used minimally invasive method for long-term enteral feeding. Identification of simple predictors for short-term mortality (up to one month) after PEG insertion is of paramount importance.

**Aim:**

We aimed to explore a simple noninvasive parameter that would predict survival following PEG insertion.

**Methods:**

We performed a retrospective study of all patients who underwent PEG insertion at the Galilee Medical Center from January 1, 2014 to December 30, 2018. We collected simple clinical and laboratory parameters and survival data and looked for predictors of short-term mortality.

**Results:**

A total of 272 patients who underwent PEG insertion were included. Sixty-four patients (23.5%) died within one month after PEG insertion compared to 208 patients (76.5%) who survived for more than one month. Univariate analysis revealed several short-term mortality-related predictors, including older age (OR 1.1, *P*=0.005), ischemic heart disease (OR 2, *P*=0.0197), higher creatinine level (OR 2.3, *P*=0.0043), and elevated CRP level and CRP-to-albumin ratio (OR 1.1, *P* < 0.0001; OR 1.0031, *P* < 0.0001, respectively). In multivariate logistic analysis, older age (OR 1.1, *P*=0.019), higher creatinine level (OR 1.6, *P*=0.074), and elevated CRP-to-albumin ratio (OR 1.1, *P*=0.002) remained significant predictors of short-term mortality after PEG insertion with an ROC of 0.7274.

**Conclusion:**

We could identify several simple parameters associated with high risk of mortality, and we recommend considering using these parameters in decision-making regarding PEG insertion. Further prospective studies are needed to validate our findings.

## 1. Introduction

Percutaneous endoscopic gastrostomy (PEG) tube insertion has become the most common method for enteral nutrition. It is mainly reserved for patients who are unable to maintain long-term adequate oral feeding of at least 2–3 weeks and for malnourished patients who are unable to satisfy their body energy requirements [1, 2]. The clinical indications for primary PEG insertion for short-term feeding are varied including dementia, dysphagia, unconsciousness, neuromuscular disorders, and patients with head and neck cancers [3]. Although PEG is considered a minimally invasive and safe interventional procedure, it is associated with short- and long-term complications including peritonitis, bleeding, aspiration, abdominal wall infection, tube leaks, tube blockage, and buried bumper syndrome [2, 4]. Moreover, several studies have reported a 30-day mortality that ranges from 3.3%–23.9% [5–7]. Therefore, the American Gastroenterological Association recommends PEG insertion in patients who are expected to survive longer than one month after the procedure [8]. Thus, the identification of specific predictors for short-term (within one month) mortality is crucial in order to correctly stratify patients who may benefit from PEG feeding.

The aim of our study was to identify clinical and laboratory predictors of short-term mortality in patients referred for PEG insertion in a tertiary medical center.

## 2. Materials and Methods

We performed a single center retrospective study of all patients over 18 years old who were scheduled for or underwent primary PEG insertion for various clinical indications at the Galilee Medical Center from January 1, 2014 to December 30, 2018. Patients who underwent primary PEG insertion were identified according to the International Classification of Diseases (ICD-9-CM).

All medical records of eligible patients were reviewed, and the following parameters were collected: demographic data (age and gender), medical history, indication for PEG insertion (dementia, stroke, anoxic brain damage, dysphagia, debilitated cancer patients, neurological degenerative disease, and cerebral palsy), and laboratory tests (alanine aminotransferase (ALT), aspartate transaminase (AST), alkaline phosphatase (ALP), gamma-glutamyl transferase (GGT), total bilirubin, creatinine, albumin, C-reactive protein (CRP), hemoglobin, platelet-to-lymphocyte ratio, neutrophil-to-lymphocyte ratio, and albumin-to-C-reactive protein ratio), as well as short-term (up to one month) and long-term (over one month) survival after PEG insertion. The primary aim of our study was to assess predictors of short-term (up to one month) mortality and long-term (more than 1 month) survival following PEG insertion. The secondary aim of our study was to characterize predictors of patient death while waiting for PEG insertion, as the maximal waiting time for PEG insertion in our center is one week. Primary comparison was performed between patients who survived more than one month after PEG insertion to patients who died within one month of the procedure.

The study protocol conforms to the ethical guidelines of the 1975 Declaration of Helsinki and was approved by the local ethics committee. Written informed consent was waived by the local ethics committee due to the retrospective noninterventional nature of the study.

## 3. Statistical Analysis

A univariate descriptive statistic was used. Data were reported as mean ± standard deviation (SD) for quantitative continuous variables and frequencies (percentages) for categorical variables. Univariate and multivariate logistic regression were used to estimate odds ratios (OR) of baseline factors, and backward selection was used to select the final model. The overall diagnostic accuracy of the scoring system was determined by a receiver operator characteristic (ROC) curve, odds ratio, and positive likelihood ratio. All analyses were carried out using the statistical analysis software (SAS Vs 9.4 Copyright (c) 2016 by SAS Institute Inc., Cary, NC, USA).

## 4. Results

### 4.1. Characteristics of the Patients' Populations with Short- and Long-Term Survivals following PEG

A total of 272 patients who underwent primary PEG insertion were identified. Sixty-four patients (23.5%) died within one month after PEG insertion (group A), compared to 208 patients (76.5%) who survived for more than one month (group B). The mean age in group A was 77.3 ± 14 years as compared to 70.4 ± 17 years in group B. Thirty-two patients of group A (11.8% of the cohort) were male, compared to 103 patients (37.8% of the cohort) in group B. The two most common indications for primary PEG insertion in groups A and B were dementia (54.7% vs. 41.3%) and stroke (43.7% vs. 37%), respectively. Baseline demographics and clinical and laboratory parameters are shown in Table 1.

### 4.2. Parameters Associated with Survival after PEG Insertion in Univariate and Multivariate Regression Analysis

In univariate regression analysis, we identified two predictors of long-term survival; higher serum albumin and hemoglobin levels were correlated with long-term survival following PEG insertion (OR 2.973, 95% CI 1.641–5.387, *P*=0.0003; OR 1.294, 95% CI 1.101–1.520, *P*=0.0018), respectively. The ROC of albumin was 0.65 with albumin values ranging from 3.3 to 3.6 gr/dl and associated with a specificity of 90–100%, sensitivity of 20%, positive predictive value of 87–100%, and negative predictive value of 25%. Similarly, the ROC curve for hemoglobin was 0.64 with hemoglobin values ranging from 12–13.6 gr% and associated with a specificity of 88–98%, sensitivity of 10–21%, positive predictive value (PPV) of 85–95%, and negative predictive value (NPV) of 25% (Tables 2 and 3). On multivariate logistic analysis, higher hemoglobin level remained positively correlated with long-term survival (OR 1.18, 95% CI 0.995–1.401, *P*=0.05).

On the other hand, several predictors were found to be correlated with short-term mortality. Older age (OR 1.1, 95% CI 1.05–1.1, *P*=0.005), ischemic heart disease (OR 2, 95% CI 1.1–3.7, *P*=0.0197), higher creatinine level (OR 2.3, 95% CI 1.3–4, *P*=0.0043), and elevated CRP level and CRP-to-albumin ratio (OR 1.1, 95% CI 1.01–1.02, *P* < 0.0001; OR 1.1, 95% CI 1.02–1.03, *P* < 0.0001, respectively). The ROC curve analysis for older age, creatinine, CRP, and CRP-to-albumin ratio were 0.63, 0.63, 0.67, and 0.68, respectively.  Table 4 shows the univariate analysis data, and Table 3 shows the specificity, sensitivity, positive predictive value (PPV), and negative predictive value (NPV) of each parameter of several cutoff point values according to ROC curve analysis.

**Figure 1:**
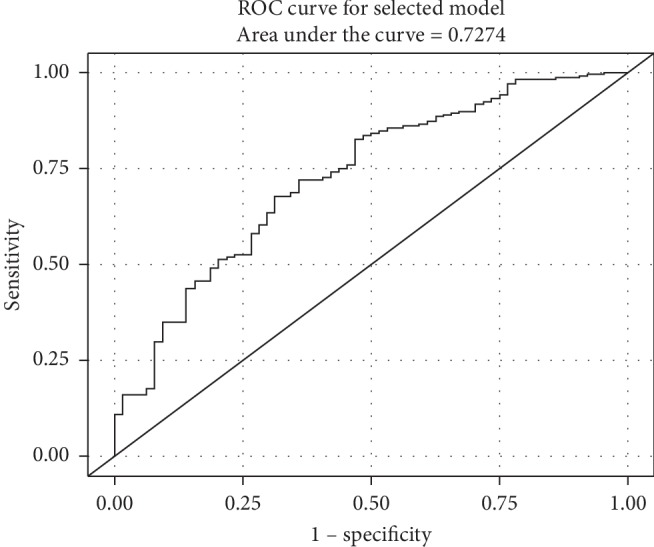
ROC curve analysis for predictors that were significant on multivariate regression analysis for short-term mortality.

On multivariate logistic analysis, older age (OR 1.1, 95% CI 1.05–1.1, *P*=0.019), higher creatinine level (OR 1.6, 95% CI 0.96–2.6, *P*=0.074), and elevated CRP-to-albumin ratio (OR 1.1, 95% CI 1.03–1.1, *P*=0.002) remained positively correlated with short-term mortality after PEG insertion with an ROC of 0.7274 (Figure 1).

## 5. Discussion

We found in our study that advanced age, poor renal function as measured by elevated creatinine values, and high CRP/albumin ratio predict short-term mortality as shown by multivariate analysis with an ROC of 0.63, 0.63, and 0.68 respectively. We also found that higher hemoglobin level was the predictor of long-term survival on multivariate analysis. In our study, short-term mortality after PEG insertion was as high as 23.5% in the cohort, similar to previous two studies where the short-term mortality rate after PEG insertion was up to 24% [6, 9]. This result is not far removed from results of a recent study reporting a short-term mortality of 27% in patients 80 years or older [10].

Identifying predictors of long-term survival or short-term mortality is one of the main concerns of practitioners who wish to offer optimal treatment to patients but would choose not to perform a “technically” successful intervention if that intervention is not likely to clinically benefit the patient. PEG tube insertion is being increasingly used for enteral nutrition, [1, 2] too often as the result of an inappropriate decision for the wrong patient. In some cases, this decision is probably influenced by the patient and family preference by cost considerations or by administrative considerations to facilitate discharge to a nursing home. Von Preyss-Friedman SM. et al. showed that physician decisions regarding PEG insertion were influenced by the patient and family preferences and by legal and cost considerations [11]. To overcome the abovementioned obstacles, we identified several simple bedside clinical and laboratory predictors that would stratify patients to PEG insertion according to their expected survival, avoiding insertion in patients with high likelihood of short-term mortality, in accordance with the American Gastroenterology Association recommendations [7].

The mean age of patients with short-term mortality was 77.3 years as compared to 70.4 years in the long-term survival group. Our results were in agreement with previously published studies where advanced age was shown to be associated with high short-term mortality [10, 12, 13]. Similarly, low concentration of albumin was shown to be associated with poor outcome and higher mortality [12, 14–16]. Moreover, several studies have demonstrated that high serum CRP levels [17, 18] and low serum albumin levels [18, 19] were associated with short-term mortality. Serum CRP-to-albumin ratio has been shown previously to be a predictor for overall survival in several cancer states [20] and in coronary artery disease [21]. Furthermore, this marker has been shown to have a prognostic role in acute pancreatitis [22]. In our study, we showed for the first time that serum CRP-to-albumin ratio is a strong predictor of short-term mortality after PEG insertion. In addition, a large, population-based study of more than 180,000 inpatients showed that renal failure was correlated with high short-term mortality [13]. Again, this correlation was repeated in our study. Therefore, these patients with high likelihood of short-term mortality can be fed by a minimally invasive and effective enteric feeding method such as nasogastric tube feeding for the 30-day period. Survivors can be then reevaluated at the end of this period for long-term enteric feeding by PEG.

On the other hand, in our study, we found that higher hemoglobin level was the only predictor of long-term survival in multivariate analysis. A low hemoglobin level of less than 11 gr/dl was shown to be associated with higher short-term mortality [12].

The limitations of our study include its retrospective nature based on patient files, with no control group. A second limitation is that the reason for death was not detailed, and it is not known whether death was related to the patient's characteristics or the PEG procedure itself. Finally, the functional status of the patients, which might affect survival [23], was not reported.

In conclusion, several predictors could affect short-term mortality, and they should be examined carefully to help stratify the patient who may gain benefit from enteral nutrition via PEG insertion. We recommend performing a prospective controlled study comparing the effect of different parameters including indication for PEG, age, hemoglobin, renal function, and albumin-to-CRP ratio on short-term mortality of two comparable groups of patients, with and without PEG, thus trying to eliminate the effect of PEG by itself on patient outcome.

## Figures and Tables

**Table 1:** Demographics and laboratory characteristics of study cohort.

Parameters	Group A (short-term mortality)	Group B (long-term survivals)
Number of patients	64	208
Age (years) (mean ± SD)	77.3 ± 14	70.4 ± 17
Gender, *N* (%)		
Male	32 (50)	32 (50)
Female	103 (49.5)	105 (50.5)
Background diseases, *N* (%)		
Diabetes mellitus	24 (37.5)	70 (33.6)
Ischemic heart disease	25 (39)	50 (24)
Congestive heart failure	11 (17.2)	21 (10.1)
Hypertension	45 (70.3)	142 (68.3)
Stroke	27 (42.2)	63 (30.3)
Indications for PEG, *N* (%)		
Dementia	35 (54.7)	86 (41.3)
Stroke	28 (43.7)	77 (37)
Anoxic brain damage	9 (14.1)	31 (14.9)
Degenerative disease	2 (3.1)	10 (4.8)
Cerebral palsy	3 (4.7)	4 (1.9)
Malignancy (head, neck, and oropharyngeal)	1 (1.6)	26 (12.5)
ALT (unit/L)	26.9 ± 28	35.5 ± 48
AST (unit/L)	36.5 ± 28.2	32.6 ± 27
ALP (unit/L)	93 ± 48	93.4 ± 49.9
Creatinine (mg/dl)	1.06 ± 1.03	0.71 ± 0.38
Albumin (g/dl)	2.65 ± 0.5	2.92 ± 0.5
Hemoglobin (g/dl)	9.68 ± 1.94	10.55 ± 1.8
CRP (mg/L)	103.7 ± 84.5	60.7 ± 61.1
CRP-to-albumin ratio	42.2 ± 39.3	22.3 ± 24

**Table 2:** Univariate analysis of parameters associated with long-term survival.

Parameter	Odds ratio	95% confidence interval	*P* value
Albumin (g/dl)	2.973	1.641–5.387	0.0003
Hemoglobin (g/dl)	1.294	1.101–1.520	0.0018

**Table 3:** Specificity, sensitivity, PPV, and NPV of several cutoff point values according to ROC curve analysis for short-term mortality and long-term survivals.

Parameter	ROC curve	Specificity (%)	Sensitivity (%)	PPV (%)	NPV (%)
Albumin (g/dl)	0.65				
3.3–3.6		90–100	10–20	87–100	25
3–3.29		74–88	25–39	83–87	27
2.6–2.9		50–68	48–72	83	29–35
2.3–2.59		28–48	76–89	80–83	38–44
<2.3		10–25	90–100	77–80	50–100

Hemoglobin (g/dl)	0.64				
11.7–13.6		81–98.5	10–27	83–95	25
9.8–11.6		63–78	33–60	83	26–33
8.5–9.7		30–58	65–87	80–83	33–41
<8.5		15–25	90–100	77–80	48–100

Creatinine (mg/dl)	0.63				
0.35–0.5		85–99	14–22	75–83	24
>0.5–0.63		61–83	26–53	83	26–29
0.65–0.84		40–59	58–79	82	30–37
0.85–0.99		30–38	82–88	80	39–42
>1		5–20	90–100	77–80	50–100

CRP (mg/L)	0.67				
1.8–17.6		90–100	20–25	90–100	24–27
>18–41		70–89	27–53	85–89	27–31
42–64		59–69	54–66	85	31–35
>73–151		30–58	71–90	80–85	38–45
>155		15–25	90–100	77–80	50–100

CRP-to-albumin ratio	0.68				
1–7		90–100	10–30	92–100	24–28
>7–17		70–88	30–60	86–89	28–35
>17–33		50–67	61–80	85	35–44
35–60		28–48	81–90	80–84	44–50
>60		10–26	91–100	77–80	55–100

**Table 4:** Univariate analysis of parameters associated with short-term mortality.

Parameter	Odds ratio	95% confidence interval	*P* value
Age	1.1	1.05–1.1	0.005
Gender male vs female	1.01	0.58–1.78	0.9461
Background diseases			
Diabetes mellitus	1.2	0.66–2.12	0.56
Ischemic heart disease	2	1.1–3.7	0.0197
Congestive heart failure	1.87	0.85–4.1	0.1182
Hypertension	1.08	0.59–2	0.7837
Stroke	1.68	0.94–2.98	0.0776
Indications of PEG			
Dementia	0.680	0.385–1.202	0.1845
Stroke	0.805	0.454–1.426	0.4570
Anoxic brain damage	1.464	0.622–3.444	0.3825
Degenerative disease	6.820	0.344–13.365	0.2079
Cerebral palsy	1.359	0.199–9.263	0.7539
Malignancy	1.077	0.353–3.284	0.8965
ALT (unit/L)	1	0.98–1	0.2872
AST (unit/L)	1.004	1.01–1.01	0.3406
ALP (unit/L)	1	0.99–1.006	0.9473
Creatinine (mg/dl)	2.3	1.3–4	0.004
CRP (mg/L)	1.1	1.01–1.02	<0.0001
CRP-to-albumin ratio	1.1	1.02–1.03	<0.0001

## Data Availability

The data are found in the gastroenterology unit at the Galilee Medical Center.

## Abbreviations

PEG: Percutaneous endoscopic gastrostomy
ALT: Alanine aminotransferase
ALP: Alkaline phosphatase
GGT: Gamma-glutamyl transferase
CRP: C-reactive protein
SD: Standard deviation
OR: Odds ratios
ROC: Receiver operator characteristics
PPV: Positive predictive value
NPV: Negative predictive value.
